# Development of Multidrug Resistant Tuberculosis in Bangladesh: A Case-Control Study on Risk Factors

**DOI:** 10.1371/journal.pone.0105214

**Published:** 2014-08-19

**Authors:** Mahfuza Rifat, Abul Hasnat Milton, John Hall, Christopher Oldmeadow, Md. Akramul Islam, Ashaque Husain, Md. Wahiduzzaman Akhanda, Bodrun Naher Siddiquea

**Affiliations:** 1 The University of Newcastle, Newcastle, New South Wales, Australia; 2 BRAC, Dhaka, Bangladesh; 3 National Tuberculosis Control Programme, Directorate General of Health Services, Dhaka, Bangladesh; 4 National Institute of Diseases of the Chest and Hospital, Dhaka, Bangladesh; University of Minnesota, United States of America

## Abstract

**Objective:**

To determine the risk factors for developing multidrug resistant tuberculosis in Bangladesh.

**Methods:**

This case-control study was set in central, district and sub-district level hospitals of rural and urban Bangladesh. Included were 250 multidrug resistant tuberculosis (MDR-TB) patients as cases and 750 drug susceptible tuberculosis patients as controls. We recruited cases from all three government hospitals treating MDR-TB in Bangladesh during the study period. Controls were selected randomly from those local treatment units that had referred the cases. Information was collected through face-to-face interviews and record reviews. Unadjusted and multivariable logistic regression were used to analyse the data.

**Results:**

Previous treatment history was shown to be the major contributing factor to MDR-TB in univariate analysis. After adjusting for other factors in multivariable analysis, age group “18–25” (OR 1.77, CI 1.07–2.93) and “26–45” (OR 1.72, CI 1.12–2.66), some level of education (OR 1.94, CI 1.32–2.85), service and business as occupation (OR 2.88, CI 1.29–6.44; OR 3.71, CI 1.59–8.66, respectively), smoking history (OR 1.58, CI 0.99–2.5), and type 2 diabetes (OR 2.56 CI 1.51–4.34) were associated with MDR-TB. Previous treatment was not included in the multivariable analysis as it was correlated with multiple predictors.

**Conclusion:**

Previous tuberculosis treatment was found to be the major risk factor for MDR-TB. This study also identified age 18 to 45 years, some education up to secondary level, service and business as occupation, past smoking status, and type 2 diabetes as comorbid illness as risk factors. National Tuberculosis programme should address these risk factors in MDR-TB control strategy. The integration of MDR-TB control activities with diabetes and tobacco control programmes is needed in Bangladesh.

## Introduction

Despite an overall decreasing incidence and mortality rate for tuberculosis (TB), multidrug resistance tuberculosis (MDR-TB) continues to be a serious threat to the current global tuberculosis control effort [1], [2]. MDR-TB is caused by bacteria that are resistant to at least isoniazid and rifampicin, the most effective anti-TB drugs for treating TB [3]. MDR-TB does not respond to standard six-month treatment with first-line anti-TB drugs; extended treatment is required involving drugs that are more toxic and more expensive [3]. Cure rate of MDR-TB is 50 to 70% which is lower than the drug-susceptible TB [4]. Failure to control MDR-TB may lead to another era with TB being regarded as a fatal disease.

Bangladesh is one of the 27 high burden countries for MDR-TB [5]. In Bangladesh, 1.4% of new tuberculosis patients, and 29% of previously treated tuberculosis patients are estimated to be MDR-TB [1]. Although the proportion of MDR-TB is still low, due to the overall high TB burden in Bangladesh the absolute number of MDR cases is quite large (estimated 1900 for new and 2300 for previously treated patients) [1]. Bangladesh is unique in that it has one of the highest population densities in the world, is one of the high burden countries for TB, but has a low prevalence of HIV [6].

Identifying the population at risk of MDR-TB is essential and may help in developing appropriate case finding strategies [7]. Previous studies identified some risk factors associated with MDR-TB, namely previous TB treatment [8], [9], [10], [11], [12], poor past compliance with treatment [12], [13], HIV infection [9], [14], younger age-group [9], [15], [16], gender [9], [13], foreign born people [9], [16], living in an urban area [15], working in health care [14], type by bacteriology and pulmonary site of TB [14], presence of cavitation in lungs [12], contact with a TB patient [11], smoking or other substance misuse [14], [17], [18], chronic renal failure [19], diabetes [20], use of other anti-microbial medicine [19], being an asylum seeker [14], living in a nursing home [14], being a prisoner [14], and hospitalization history [21]. Inappropriate medical management, absence of directly observed treatment, lack of uniformity between public and private sectors, limited or interrupted drug supply, poor quality and widespread availability of anti-tuberculosis drugs, were also reported as important causes associated with MDR-TB [10], [22], [23]. However, findings related to some risk-factors such as HIV status [10], [24], age group [10] and gender of the patients [8], [9], [13] differed. Moreover, study designs varied widely, some findings were based on small sample sizes and some came from drug resistance surveys.

Characteristics of MDR-TB patients have not been systematically explored in Bangladesh. Flora et al. conducted a study in 2010 that recruited a small number of purposively selected participants [25], making it impossible to generalise the findings of the study. There were also a few discrepancies between the presented results and the conclusions drawn. The authors reported that only 30 (22.1%) MDR-TB patients and seven (4.6%) drug sensitive TB patients had a previous history of tuberculosis. However, they included the total sample in the analysis to test the factors related to past illness, such as “Course of treatment” and “Directly observed treatment” [25]. It is not clear whether they were looking for the effects of current or previous treatment episodes. The National Tuberculosis Control Programme (NTP) Bangladesh started the MDR-TB programme in 2008 and gradually expanded its services in subsequent years [26]. At the time of the previously conducted study the MDR-TB programme was still evolving.

This is the context for our case-control study that explores the factors associated with MDR-TB. We compared the backgrounds and histories of MDR and drug-susceptible TB patients. All consenting MDR-TB patients aged between 18 and 65 years who were treated at one of the three government hospitals responsible for MDR-TB treatment in the country during the study period between September 2012 and April 2013 were included in the study.Controls were selected randomly from those local treatment units that had referred the cases.

## Methods

### Ethics considerations

The study was approved by the Human Research Ethics Committee (HREC) of the University of Newcastle (UoN), Australia and the Bangladesh Medical Research Council (BMRC), Dhaka, Bangladesh. An information sheet describing the purpose of the study and the individuals' rights as study participants was handed to the participants to read. For individuals with inadequate literacy, the information sheet was read out by the interviewers. Written informed consent was then obtained from each person. A thumb impression was obtained from those who were unable to sign the consent form. All patients had been treated through the National TB Control Programme (NTP).

### Study population and design

Patients were recruited from central, district and sub-district level government hospitals and Non-governmental organization (NGO) clinics in rural and urban Bangladesh. This case-control study includes 250 MDR-TB patients as cases and 750 drug-sensitive TB patients as controls. We designed the study to have 80% power to detect at least a 10% difference in the prevalence of any of our exposure variables at 5% significance threshold, assuming prevalence in the controls of 40% (with greater power and smaller effects detectable for exposures with lower control prevalence). This sample allowed us to accommodate the multivariable analysis for multiple factors.

MDR-TB patients aged between 18 to 65 years who gave their informed consent were included in the study. Patients who received treatment for MDR-TB following the criteria of the national guidelines of the National Tuberculosis Control Programme (NTP) were classified as MDR-TB. The NTP has recently adopted automated real time PCR (Xpert MTB/RIF) as the diagnostic tool of MDR-TB patients. Culture and Drug Sensitivity Testing (DST) and Line probe assays were also used [27]. Xpert MTB/RIF diagnoses only Rifampicin resistance. Patients who are resistant to Rifampicin are generally also resistant to Isoniazid (another first-line drug) as well. Mono-resistance to Rifampicin is fairly uncommon (0.2% and 0.4% among new and previously treated patients, respectively), as shown by a recent drug resistance survey (DRS) conducted in Bangladesh [28]. Controls were drug susceptible TB patients aged 18 to 65 years, diagnosed through sputum smear microscopy or other investigations (X-ray, FNAC, and Biopsy) as per national guidelines who would respond to the standard combination of drugs. In this paper we will refer to those as non-MDR-TB patients.

We excluded patients who were not within the eligible age group or had any serious illness requiring admission to the Intensive Care Unit (ICU), recent surgery or any medical emergency that needs continuous observation.

### Data collection

MDR-TB patients from all over Bangladesh are referred to one of the three government hospitals (to the national hospital in Dhaka or a regional hospital in either Chittagong or Rajshahi). We consecutively recruited all eligible MDR-TB patients who were admitted from September 2012; recruitment ceased in April 2013 when the target of 250 cases was reached.

We recruited three controls per case from the local tuberculosis treatment unit from where the case was referred. The hospitals that were providing MDR-TB treatment were receiving patients referred by the various treatment units from rural and urban Bangladesh. Each TB patient is assigned a unique TB registration number as a routine practice. Treatment registration numbers of the tuberculosis patients, who were diagnosed during the specified period i.e. during the same month that MDR-TB was diagnosed, were listed. Three controls per MDR-TB case were randomly selected from this list at the treatment unit.

Trained investigators collected information from the study participants using a pretested questionnaire through a face-to-ace interview and review of records. All the investigators received training on data collection procedures for one week. The NTP has its inbuilt quality control mechanism for diagnosis of MDR-TB patients through a laboratory based in Antwerp, Belgium. Diagnosis of drug-sensitive tuberculosis through microscopy is under an external quality assessment (EQA) network at country level.

### Statistical analysis

A data entry template was used and data was validated by a series of logical checks. Summary statistics and tables were produced from cleaned and acceptable data. We compared participant characteristics between MDR-TB cases and controls using Student t-tests for continuous measures, and Chi-square (χ^2^) tests for categorical measures. Associations between participant characteristics and MDR-TB status were assessed using both unadjusted and multivariable logistic regression models. We had sufficient MDR-TB cases to include the following variables in the multivariable model without risk of over-fitting: age, gender, educational status, occupation, history of health care related work, monthly household income, living conditions (number of persons per room), BCG vaccination status, contact with other TB patients, smoking, substance misuse (alcohol or drug addiction), type 2 diabetes as co-morbidity, and hospitalization history. We included all variables to the initial multivariable model and variables were removed from this model if the Likelihood ratio test was not significant at 5% and the coefficients of the remaining variables did not change by more than 15% (indicating no evidence of confounding). Collineartiy was assessed through inspecting variance inflation factors and assessing pair-wise Chi-Square tests. Data analysis was carried out using Stata statistical software version 12 (StataCorp LP, TX, USA).

## Results

### Socio-demographic and clinical characteristics

The study included 250 MDR-TB and 750 non-MDR-TB patients representing all seven divisions of Bangladesh. Mean age of participants was 37 years and 61% were male. About half of the participants had some education at secondary level or below and a median monthly income of 10000 Bangladeshi taka (129 USD approximately).

Details of Socio-demographic and clinical characteristics are shown in Table 1.

**Table 1:** Socio-demographic and clinical characteristics of the study participants.

Variables	Case	Control	Total	p^a^
**Age**				0.0001
Mean	33.9	37.9	36.9	
Median	30	35	35	
SD	12.3	14.1	13.8	
**Sex**				0.027
Male	167 (66.8%)	442 (58.9%)	609 (60.9%)	
Female	83 (33.2%)	308 (41.1%)	391 (39.1%)	
**Education**				<0.0001
None	55(22%)	298 (39.7%)	353 (35.3%)	
Secondary and below	175 (70%)	398 (53.1%)	573 (57.3%)	
Higher secondary and above	20 (8%)	54 (7.2%)	74 (7.4%)	
**Occupation**				
None	9 (3.6%)	58 (7.7%)	67 (6.7%)	<0.0001
Service	74 (29.6%)	135 (18%)	209 (20.9%)	
Others b	108 (43.2%)	447 (59.6%)	555 (55.5%)	
Business	46 (18.4%)	79 (10.5%)	125 (12.5%)	
Transport worker	13 (5.2%)	31 (4.1%)	44 (4.4%)	
**Income (BDT)** c				
Mean	13066.0	11820.2	12132.0	0.1206
Median	10000	10000	10000	
SD	11016.3	10965.8	13.8	
**Person living per room**				0.069
Four or less	215 (86%)	676 (90.1%)	891 (89.1%)	
More than four	35 (14%)	74 (9.9%)	109 (10.9%)	
**Weight (killogram)**				
Mean	42.5	44.6	44.0	0.002
Median	41.0	44.0	43.0	
SD	9.7	9.1	9.3	
**BCG vaccination status**				0.056
Absent	123 (49.2%)	317 (42.3%)	440 (44%)	
Present	127 (50.8%)	433 (57.7%)	560 (56%)	
**Previous history of TB treatment**				<0.0001
No	5 (2%)	702 (93.6%)	707 (70.7%)	
Yes	245 (98%)	48 (6.4%)	293 (29.3%)	
**Cavitation in chest X-ray** d				<0.0001
Absent	136 (90.7%)	330 (98.2%)	466 (95.9%)	
Present	14 (9.3%)	6 (1.8%)	20 (4.1%)	
**History of Health care work**				0.144
Absent	246 (98.4%)	722 (96.3%)	968 (96.8%)	
Present	4 (1.6%)	28 (3.7%)	32 (3.2%)	
**Contact of TB patient**				0.496
Absent	153 (61.2%)	477 (63.6%)	630 (63%)	
Present	97 (38.8%)	273 (36.4%)	370 (37%)	
**Smoking status**				<0.0001
Never smoked	125 (50%)	409 (54.5%)	534(53.4%)	
Current smoker	1(0.4%)	82 (10.9%)	83 (8.3%)	
Past smoker	124 (49.6%)	259 (34.5%)	383 (38.3%)	
**Substance misuse**				0.013
Absent	213(85.2%)	681 (90.8%)	894 (89.4%)	
Present	37 (14.8%)	69 (9.2%)	106 (10.6%)	
**Type-2 Diabetes**				<0.0001
Absent	216 (86.4%)	701 (93.5%)	917 (91.7%)	
Present	34 (13.6%)	49 (6.5%)	83 (8.3%)	
**Kidney disease**				1.000
Absent	248 (99.2%)	745 (99.3%)	993 (99.3%)	
Present	2 (0.8%)	5 (0.7%)	7 (0.7%)	
**Other disease** e				0.831
Absent	242 (96.8%)	728 (97.1%)	970 (97%)	
Present	8 (3.2%)	22 (2.9%)	30 (3%)	
**Hospitalization history** f				0.194
Absent	246 (98.4%)	724 (96.7%)	970 (97.1%)	
Present	4 (1.6%)	25 (3.3%)	29 (2.9%)	

a P is the Probability of t-test or Chi-square (χ ^2^) tests. Fisher's exact Chi-square (χ ^2^) test was used for history of health care work, kidney disease, other disease, smoking status and hospitalization history.

b ‘Others’ subgroup under ‘Occupation’ includes housewife and self-employed small works.

c BDT: Bangladeshi currency.

d Cavitation related information was not available in 51% of the participants.

e Other disease included hypertension, heart diseases, asthma, chronic obstructive pulmonary diseases and chronic dysentery.

f Hospitalization history had one missing value.

### Risk factors for MDR-TB

#### Univariate analysis

Previous history of tuberculosis treatment was a major contributing factor to MDR-TB (OR 716.6, 95% CI 282.1–1820.8). In total, 29.3% of participants had a history of previous tuberculosis treatment that was 98% of the MDR-TB and 6.4% of non-MDR-TB patients. MDR-TB patients were more likely to be male, aged between 18 and 45, educational level of secondary and below or higher secondary and above, have an occupation in service or business or transport work, are a smoker, have a history of substance misuse or type 2 diabetes (Table-2).

**Table 2:** Univariate logistic regression analysis on factors related to Multidrug Resistant Tuberculosis (MDR-TB).

Variables	Odds ratio	Confidence Interval a	p b
**Previous history of TB Treatment**			
No	1.00		
Yes	716.63	282.1–1820.8	<0.0001
**Gender**			
Female	1.00		
Male	1.4	1.0–1.9	0.028
**Age-group**			
More than 45 years	1.00		
18 to 25 years	1.97	1.3–3.0	0.001
26 to 45 years	2.06	1.4–3.0	<0.0001
**Education**			
None	1.00		
Secondary and below	2.38	1.7–3.3	<0.0001
Higher secondary and above	2.01	1.1–3.6	0.02
**Occupation**			
None	1.00		
Service	3.53	1.7–7.5	0.001
Others c	1.56	0.7–3.2	0.236
Business	3.75	1.7–8.3	0.001
Transport worker	2.70	1.0–7.0	0.041
**Smoking status**			
Never smoked	1.00		
Current smoker	0.04	0.05–0.3	0.001
Past smoker	1.57	1.2–2.1	0.003
**Substance misuse**			
No	1.00		
Yes	1.71	1.1–2.6	0.014
**Type-2 Diabetes**			
No	1.00		
Yes	2.25	1.4–3.6	0.001

a Confidence interval at 95% level.

b p is the p value of Wald test statistic.

c ‘Others’ subgroup under ‘Occupation’ includes housewife and self-employed small works.

Only the significant variables are shown in the table (significance level at 0.05).

#### Multivariable analysis

We removed previous treatment from the multivariable model since the variance inflation factors were high and it had a high degree of association with many of the variables in the model. The variables showing strong association with previous treatment included age (Chi-square 7.2,df 2; p 0.027), educational status (Chi-square 15.3, df 2; p <0.0001), occupation (Chi-square 22.4, df 4; p <0.0001), history of health care related work (Chi-square 6.3, df 1; p 0.01), monthly household income (Chi-square 15.0, df 4; p 0.005), smoking (Chi-square 29.2, df 2; p < 0.0001), substance misuse (Chi-square 7.3, df 1; p 0.007) and type 2 diabetes as co-morbidity (Chi-square 8.7, df 1; p 0.003).

For the final multivariable model, we found that age group, educational status, occupation, smoking status, and type 2 diabetes were significantly associated with MDR-TB (Table-3).

**Table 3:** Multivariable analysis on factors related to Multidrug Resistance Tuberculosis (MDR-TB).

Predictor	Adjusted Odds ratio	Confidence Interval a	p^b^ (Wald)	p^c^ (lrt)
**Age group**				0.0325
More than 45 years	1.00			
18 to 25 years	1.77	1.07–2.93	0.027	
26 to 45 years	1.72	1.12–2.66	0.013	
**Education**				0.0026
None	1.00			
Secondary and below	1.94	1.32–2.85	0.001	
Higher secondary and above	1.83	0.92–3.65	0.086	
**Occupation**				
None	1.00			0.002
Service	2.88	1.29–6.44	0.010	
Others d	1.65	0.76–3.55	0.203	
Business	3.71	1.59–8.66	0.002	
Transport worker	2.71	0.95–7.72	0.063	
**Smoking status**				**<0.0001**
Never smoked	1.00			
Current smoker	0.04	0.005–0.29	0.002	
Past smoker	1.58	0.99–2.50	0.053	
**Type-2 Diabetes**				0.0006
Absent	1.00			
Present	2.56	1.51–4.34	0.001	

a Confidence interval at 95% level.

b p (Wald) is the p value of Wald test statistic.

c p (lrt) is the p value corresponding to the Likelihood ratio test statistic.

d “Others” subgroup under “Occupation” includes housewife and self-employed small works.

Only the significant variables in multivariable model are shown in the table (significance level 0.05).

## Discussion

Multidrug resistance is more commonly reported among previously treated tuberculosis patients than in new tuberculosis patients, globally as well as in Bangladesh [1]. Our study showed that most MDR-TB patients (98%) had a history of previous tuberculosis treatment, in line with other studies [8], [9], [10], [11], [12], [17]. In a systematic review of risk factors conducted in Europe, previous treatment history of TB was the strongest determinant of MDR-TB in Europe and the pooled risk of MDR-TB was 10.23 times higher in previously treated patients than in patients without prior treatment [9]. Previous treatment as a risk factor helped in developing the MDR-TB case finding strategy during the introduction of MDR-TB programmes [7]. Drug sensitivity testing is not routinely done on all TB patients in Bangladesh due to the large number of patients diagnosed each year [27]. Recent national guidelines recommend that previously treated patients, new TB patients with treatment failure, and people in contact with MDR-TB patients, are referred for MDR-TB testing. In addition, patients with delayed response in treatment, or with smear-negative or extra-pulmonary TB that does not improve clinically, with relapse or who receive treatment after default, or who have HIV, are tested for MDR-TB in Bangladesh [27].

In our study, being between 18 and 45 years of age was associated with an increased risk for MDR-TB, similar to what was reported in another study conducted in Hong Kong [16]. Another study conducted in Bangladesh found that patients under 40 years are more likely to develop MDR-TB, based on univariate findings, although this association was weak in the multivariable model (OR 0.87; 95% CI 0.40–1.93 and OR 0.87 CI 0.33–2.33 for age-groups 21 to 30 years and 31 to 40 years, respectively) [25]. Other studies conducted in Shanghai and Spain found that the greatest risk of MDR-TB was associated with age 35 to 59 [15] and 45 to 65 years [17], respectively. Although the range varies, being below 65 years is associated with developing MDR-TB, as reported in multi-country reviews [9], [10]. Younger people are more likely to come in contact with MDR-TB as they are more mobile and active compared to the older age group through their involvement in work or study [16]. They may also find it difficult to take regular supervised medicine due to conflicting work times, which results in poor treatment adherence. Another explanation for the greater risk in younger age groups may be that Rifampicin was introduced in recent decades and many elderly people may not have been exposed to it [16]. These explanations may not be applicable to primary drug resistance that is transmitted. In our study only five (2%) MDR-TB patients did not have any history of previous treatment, in line with the low level of primary resistance in a recent drug resistance survey, where 1.4% of MDR-TB patients did not have a previous diagnosis, compared to 28% of previously treated patients [28].

A number of occupations such as those associated with services and business were more likely to be linked with MDR-TB compared to non-working individuals. Occupation as transport workers, another highly mobile group, was associated with MDR-TB if examined alone, although we did not observe any difference after adjusting for other factors. This study did not show any association with health care as an occupation, which was found to be associated with MDR-TB in another study [14]. Patients with some educational qualification were more likely to develop MDR-TB than patients with no formal education or from the highest educational group.

Type 2 Diabetes is known to be a risk factor for TB [29] and is linked to MDR-TB in our and other studies [20]. It may affect TB treatment outcome and disease presentation [29], leading to failed treatment, although this is not always the case [30]. Impaired immunity due to diabetes may increase susceptibility to infection with drug resistant strains [20]. Bangladesh is facing the dual burden of communicable and non-communicable diseases. The prevalence of Diabetes mellitus has increased from 2.3% in 1999 to 7.9% in 2009 [31]. The relationship between MDR-TB and diabetes could be addressed by treating diabetic patients with tuberculosis within a collaborative framework [32]. In our study, the diabetes status was self-reported by the patients. Further studies using a screening method for diabetes status need to be conducted.

MDR-TB patients were more likely to be past tobacco smokers in our study. Although current smokers were less likely to have MDR-TB compared to non-smokers, this may be a result of MDR-TB patients quitting smoking on diagnosis. Smoking is one of the main determinants for TB and some studies showed an association with acquired drug resistance [18]. Another study showed that smoking is a predictor for delayed response to treatment [33]. Tobacco control efforts have been initiated in Bangladesh in recent years, including some piloting of its integration with tuberculosis services [34]. Our finding suggests that TB and tobacco control efforts need to be sustained to control TB overall as well as MDR-TB. Intravenous drug use was a risk factor for MDR-TB in another study [14]. Drug or alcohol misuse was not a significant cause of MDR-TB in our study, after adjusting for other factors.

Males were more likely to have MDR-TB than females in some settings [9] whereas the opposite was true in others [8], [13]. Gender was not a risk factor in our study. Although contact with TB patients was found to be associated with MDR-TB in other studies [11], [23], [35], we did not observe any association. Neither did we observe any effect of income, crowding status expressed as persons per room, vaccination status (BCG), history of hospitalization within seven months, and kidney disease. Overall, 56% of our participants were BCG vaccinated in their childhood. Recent BCG coverage among children has increased remarkably in Bangladesh and has reached almost 98% [36].

In this study we focused on hospital based cases, as a population-based risk factor study was not feasible for MDR-TB. However, our cases are likely to be representative of MDR-TB patients in Bangladesh as we recruited from all three Government TB hospitals which treat most of the MDR-TB patients in the country. We recruited the controls from the population rather than from MDR-TB hospitals to ensure they were representative of non-MDR patients. In Bangladesh, most TB patients are treated within the NTP designated DOTS centres, and current case notification rate in Bangladesh is 68% [1]. We could not assess HIV status as a risk factor in our study. However, Bangladesh is a low burden country for HIV [6]. It was not feasible to confirm drug susceptibility using Drug Sensitivity Testing (DST) of the controls, as only high-risk patients are routinely tested, and we did not have the funds for this.

## Conclusion

Previous tuberculosis treatment was found to be the major risk factor for MDR-TB. This study also identified the following as risk factors for MDR-TB: age 18 to 45 years, some education up to secondary level, service and business as occupation, past smoking status, and type 2 diabetes as comorbid illness. These risk factors should be addressed in the strategy for MDR-TB control. The NTP of Bangladesh is reliant on multi-sectoral involvement to address all risk factors and can advocate for these issues in order to improve control of MDR-TB. The integration of MDR-TB control activities with diabetes and tobacco control programmes would be a good place to start these collaborative efforts.

## References

[1] World Health Organization (2013) Global Tuberculosis Report 2013. Geneva

[2] World Health Organization (2011) Towards the universal access to diagnosis and treatment of multidrug-resistant and extensively drug-resistant tuberculosis by 2015,WHO progress report. Geneva

[3] Caminero JA, editor (2013) Guidelines for Clinical and Operational Management of Drug-Resistant Tuberculosis. Paris, France: International Union Against Tuberculosis and Lung Disease.

[4] World Health Organization (2011) The Global Plan to Stop TB, Transforming the Fight towards Elimination of Tuberculosis. Geneva

[5] World Health Organization (2008) Anti-Tuberculosis Drug Resistance in the World, 4th Global Report. Geneva

[6] UNAIDS (2013) Global Report, UNAIDS Report on the Global AIDS Epidemic 2013. Geneva

[7] Caminero JA (2010) Multidrug-resistant tuberculosis: epidemiology, risk factors and case finding. Int J Tuberc Lung Dis 14: 382–390. 20202293

[8] Lomtadze N, Aspindzelashvili R, Janjgava M, Mirtskhulava V, Wright A, et al (2009) Prevalence and risk factors for multidrug-resistant tuberculosis in the Republic of Georgia: a population-based study. Int J Tuberc Lung Dis 13: 68–73. 19105881 PMC2645031

[9] Faustini A, Hall AJ, Perucci CA (2006) Risk factors for multidrug resistant tuberculosis in Europe: a systematic review. Thorax 61: 158–163. 16254056 10.1136/thx.2005.045963 PMC2104570

[10] Espinal MA, Laserson K, Camacho M, Fusheng Z, Kim SJ, et al (2001) Determinants of drug-resistant tuberculosis: analysis of 11 countries. Int J Tuberc Lung Dis 5: 887–893. 11605880

[11] Diande S, Sangare L, Kouanda S, Dingtoumda BI, Mourfou A, et al (2009) Risk factors for multidrug-resistant tuberculosis in four centers in Burkina Faso, West Africa. Microb Drug Resist 15: 217–221. 19728781 10.1089/mdr.2009.0906

[12] Sharma SK, Turaga KK, Balamurugan A, Saha PK, Pandey RM, et al (2003) Clinical and genetic risk factors for the development of multi-drug resistant tuberculosis in non-HIV infected patients at a tertiary care center in India: a case-control study. Infect Genet Evol 3: 183–188. 14522182 10.1016/s1567-1348(03)00086-8

[13] Ejaz M, Siddiqui AR, Rafiq Y, Malik F, Channa A, et al (2010) Prevalence of multi-drugresistant tuberculosis in Karachi, Pakistan: identification of at risk groups. Trans R Soc Trop Med Hyg 104: 511–517. 20427065 10.1016/j.trstmh.2010.03.005

[14] Casal M, Vaquero M, Rinder H, Tortoli E, Grosset J, et al (2005) A case-control study for multidrug-resistant tuberculosis: risk factors in four European countries. Microb Drug Resist 11: 62–67. 15770097 10.1089/mdr.2005.11.62

[15] Shen X, DeRiemer K, Yuan ZA, Shen M, Xia Z, et al (2009) Drug-resistant tuberculosis in Shanghai China, 2000-2006: prevalence, trends and risk factors. Int J Tuberc Lung Dis 13: 253–259. 19146756 PMC4486066

[16] Law WS, Yew WW, Chiu Leung C, Kam KM, Tam CM, et al (2008) Risk factors for multidrug-resistant tuberculosis in Hong Kong. Int J Tuberc Lung Dis 12: 1065–1070. 18713506

[17] Suarez-Garcia I, Rodriguez-Blanco A, Vidal-Perez JL, Garcia-Viejo MA, Jaras-Hernandez MJ, et al (2009) Risk factors for multidrug-resistant tuberculosis in a tuberculosis unit in Madrid, Spain. Eur J Clin Microbiol Infect Dis 28: 325–330. 18830725 10.1007/s10096-008-0627-y

[18] World health Organization, International Union Against Tuberculosis and Lung Disease (2007) A WHO/The Union Monograph on TB and Tobacco control, Joining efforts to control two related global epidemics. Geneva

[19] Kim HR, Hwang SS, Kim EC, Lee SM, Yang SC, et al (2011) Risk factors for multidrug-resistant bacterial infection among patients with tuberculosis. J Hosp Infect 77: 134–137. 20850896 10.1016/j.jhin.2010.07.004

[20] Fisher-Hoch SP, Whitney E, McCormick JB, Crespo G, Smith B, et al (2008) Type 2 diabetes and multidrug-resistant tuberculosis. Scand J Infect Dis 40: 888–893. 18728934 10.1080/00365540802342372 PMC2887603

[21] Andrews JR, Shah NS, Weissman D, Moll AP, Friedland G, et al (2010) Predictors of multidrug-and extensively drug-resistant tuberculosis in a high HIV prevalence community. PLoS One 5: e15735. 21209951 10.1371/journal.pone.0015735 PMC3012092

[22] Zaman K, Rahim Z, Yunus M, Arifeen S, Baqui AH, et al (2005) Drug resistance of Mycobacterium tuberculosis in selected urban and rural areas in Bangladesh. Scand J Infect Dis 37: 21–26. 15764186 10.1080/00365540410026095

[23] Farmer P, Bayona J, Becerra M (2001) Multidrug resistant tuberculosis and the need for biosocial perspectives. Int J Tuberc Lung Dis 5: 885–6. 11605879

[24] Suchindran S, Brouwer ES, Van Rie A (2009) Is HIV infection a risk factor for multi-drug resistant tuberculosis? A systematic review. PLoS One 4: e5561. 19440304 10.1371/journal.pone.0005561 PMC2680616

[25] Flora MS, Amin MN, Karim MR, Afroz S, Islam S, et al (2013) Risk factors of multi-drug-resistant tuberculosis in Bangladeshi population: a case control study. Bangladesh Medical Research Council Bulletin 39: 34–41. 23923410 10.3329/bmrcb.v39i1.15808

[26] National TB Control Programme DGoHS, Bangladesh (2012) Tuberculosis Control in Bangladesh, Annual Report. Dhaka

[27] National TB Control Programme DGoHS, Bangladesh (2012) Operation manual for management of Multidrug-resistant TB(MDR TB). 2nd ed. Dhaka.

[28] National TB Control Programme DGoHS, Bangladesh (2013) First Bangladesh National Tuberculosis Drug Resistance Survey 2010–2011. Dhaka

[29] Dooley KE, Chaisson RE (2009) Tuberculosis and diabetes mellitus: convergence of two epidemics. Lancet Infect Dis 9: 737–746. 19926034 10.1016/S1473-3099(09)70282-8 PMC2945809

[30] Magee MJ, Bloss E, Shin SS, Contreras C, Huaman HA, et al (2013) Clinical characteristics, drug resistance, and treatment outcomes among tuberculosis patients with diabetes in Peru. International Journal of Infectious Diseases 17: e404–412. 23434400 10.1016/j.ijid.2012.12.029 PMC8324016

[31] Bhowmik B, Afsana F, My Diep L, Binte Munir S, Wright E, et al (2013) Increasing prevalence of type 2 diabetes in a rural bangladeshi population: a population based study for 10 years. Diabetes Metab J 37: 46–53. 23439676 10.4093/dmj.2013.37.1.46 PMC3579152

[32] World Health Organization, International Union against Tuberculsois and Lung Diseases (2011) Collaborative framework for care and control of tuberculosis and diabetes. . Geneva. 26290931

[33] Qazi F, Khan U, Khowaja S, Javaid M, Ahmed A, et al (2011) Predictors of delayed culture conversion in patients treated for multidrug-resistant tuberculosis in Pakistan. Int J Tuberc Lung Dis 15: 1556–1559. 22008773 10.5588/ijtld.10.0679 PMC6203961

[34] Siddiquea BN, Islam MA, Bam TS, Satyanarayana S, Enarson DA, et al (2013) High quit rate among smokers with tuberculosis in a modified smoking cessation programme in Dhaka, Bangladesh. Public Health Action 3: 243–246. 26393038 10.5588/pha.13.0051 PMC4463131

[35] Brewer TF, Choi HW, Seas C, Krapp F, Zamudio C, et al (2011) Self-reported risks for multiple-drug resistance among new tuberculosis cases: implications for drug susceptibility screening and treatment. PLoS ONE 6: e25861. 22022459 10.1371/journal.pone.0025861 PMC3194818

[36] National Institute of Population Research and Training, Mitra and Associates, MEASURE DHS II (2012) Bangladesh Demographic and Health Survey 2011. Dhaka

